# Editorial: Advanced nanotechnology for reactive oxygen species-mediated therapies

**DOI:** 10.3389/fmolb.2022.1000113

**Published:** 2022-08-19

**Authors:** Chun Gwon Park, Wonhwa Lee, Dong-Hyun Kim, Fangyuan Li, Wooram Park

**Affiliations:** ^1^ Department of Biomedical Engineering, SKKU Institute for Convergence, Sungkyunkwan University, Suwon, South Korea; ^2^ Department of Intelligent Precision Healthcare Convergence, SKKU Institute for Convergence, Sungkyunkwan University, Suwon, South Korea; ^3^ Department of Chemistry, Sungkyunkwan University, Suwon, South Korea; ^4^ Department of Radiology, Feinberg School of Medicine, Northwestern University, Chicago, IL, United States; ^5^ Department of Biomedical Engineering, University of Illinois at Chicago, Chicago, IL, United States; ^6^ Department of Biomedical Engineering, McCormick School of Engineering, Evanston, IL, United States; ^7^ Robert H. Lurie Comprehensive Cancer Center, Chicago, IL, United States; ^8^ College of Pharmaceutical Sciences, Institute of Pharmaceutics, Zhejiang University, Hangzhou, China; ^9^ College of Pharmaceutical Sciences, Hangzhou Institute of Innovative Medicine, Zhejiang University, Hangzhou, China; ^10^ Department of Integrative Biotechnology, College of Biotechnology and Bioengineering, Sungkyunkwan University, Suwon, South Korea

**Keywords:** reactive oxygen species (ROS), nanotechnology, disease treatment and diagnosis, pyroptosis, aging

Reactive oxygen species (ROS) are oxygen-containing molecules representing high chemical reactivity (Mattila et al., 2015; Krumova and Cosa, 2016). Due to their high reactivity, ROS are generally known to damage nucleic acids, proteins, and lipids, leading to a variety of inflammations, aging, and degenerative diseases (Brieger et al., 2012). However, ROS also serve crucial physiological functions (Dröge, 2002). The level of ROS determines how precisely the body regulates immunological responses (Seifried et al., 2007; Wink et al., 2011). Our body needs an appropriate inflammatory response from ROS to defend itself against viruses and cancer (Chew and Park, 2004; Seifried et al., 2007). The ROS level also significantly influences stem cell differentiation (Yanes et al., 2010; Morimoto et al., 2013; Li et al., 2017). Stem cell differentiation is actively induced when ROS levels are acceptable, whereas differentiation of stem cells is inhibited when ROS levels are too high.

It is essential to accurately detect and measure ROS *in vivo* in order to understand the molecular mechanism underlying its level (Woolley et al., 2013; McMurray et al., 2016). On the basis of this, it will be able to create a novel notion of disease treatment if ROS can be accurately managed. Research that can precisely detect and control ROS is currently in progress, thanks to the advancement of nanotechnology (Wang et al., 2019; Yang et al., 2019). This Research Topic has dealt with the latest ROS-related research.

On this Research Topic, four articles were submitted by 25 authors from various scientific disciplines. Both original articles and reviews are found in this Research Topic.

In this review, Hu et al. describes the current state of platinum-induced peripheral neuropathy (PIPN)—a condition frequently observed in patients receiving platinum-based drugs. The authors illustrate in detail possible mechanisms of PIPN related to the overproduction of ROS and also outline various strategies currently being employed to treat PIPN. The accumulation of platinum in the dorsal root ganglia and the role of platinum-induced ROS in cellular regulation is highlighted as noteworthy aspects of PIPN. Furthermore, this review describes the most recent research in nanotechnology-based approaches to overcome PIPN and other problems traditionally associated with the use of platinum drugs.

Yang et al. described the association between aging-related diseases and accumulation of ROS. They explained a superoxide dismutase (SOD), an important enzyme in defending against oxidative damage *in vivo*, and focused the importance of maintaining appropriate ROS levels. Here, the SOD@MSN nanosystem was developed through immobilization of SOD in MSN for efficient delivery of SOD. Finally, the authors demonstrated the potential of SOD@MSN as an anti-aging treatment by presenting the result of effective internalization and protection from external stress.

In the research article, Yu et. al. presents an approach to treat gastric cancer using a combination of sonodynamic therapy and chemotherapy. The sonosensitizer (Chlorin e6) and drug (*i.e,* tirapazamine) were both loaded into ZIF-8, a zinc-based metal organic framework nanoparticle, and then coated with the cytomembrane to target the cancer cells. This nanoparticle exhibited increased cellular uptake owing to the cytomembrane coating, and produced a significant amount of ROS in an ultrasound stimulation *in vitro*. When it was administered to tumor models *in vivo*, this combination therapy was able to significantly inhibit tumor growth compared to the sonosensitizer only or drug only groups. Further investigations revealed that such cytotoxic effects were the result of nanoparticles-mediated pyroptosis.

The review by Maddheshiya and Nara addresses the roles of composite nanozymes as catalytic and/or pro-oxidative agents, hence its applications as therapeutics. In this review, the authors focus on introducing recent attempts in designing composite nanozymes to enhance their ROS-generating or other catalytic potentials. Various composite nanozymes mimicking the activities of natural enzymes are introduced as well as those with pro-oxidative potentials. The authors also illustrate key parameters that should be considered in designing pro-oxidative nanozymes. Furthermore, recent applications of composite nanozymes in ROS-mediated therapies as antibacterial, anti-biofilm, and antitumor agents are reviewed in detail as well.

Future hope for the detection and treatment of a variety of incurable diseases is projected to come from nanotechnology-based ROS detection and treatments.
