# Corrigendum: Editorial: Advanced nanotechnology for reactive oxygen species‐mediated therapies

**DOI:** 10.3389/fmolb.2022.1046852

**Published:** 2022-10-13

**Authors:** Chun Gwon Park, Wonhwa Lee, Dong-Hyun Kim, Fangyuan Li, Wooram Park

**Affiliations:** ^1^ Department of Biomedical Engineering, SKKU Institute for Convergence, Sungkyunkwan University, Suwon, South Korea; ^2^ Department of Intelligent Precision Healthcare Convergence, SKKU Institute for Convergence, Sungkyunkwan University, Suwon, South Korea; ^3^ Department of Chemistry, Sungkyunkwan University, Suwon, South Korea; ^4^ Department of Radiology, Feinberg School of Medicine, Northwestern University, Chicago, IL, United States; ^5^ Department of Biomedical Engineering, University of Illinois at Chicago, Chicago, IL, United States; ^6^ Department of Biomedical Engineering, McCormick School of Engineering, Evanston, IL, United States; ^7^ Robert H. Lurie Comprehensive Cancer Center, Chicago, IL, United States; ^8^ College of Pharmaceutical Sciences, Institute of Pharmaceutics, Zhejiang University, Hangzhou, China; ^9^ College of Pharmaceutical Sciences, Hangzhou Institute of Innovative Medicine, Zhejiang University, Hangzhou, China; ^10^ Department of Integrative Biotechnology, College of Biotechnology and Bioengineering, Sungkyunkwan University, Suwon, South Korea

**Keywords:** reactive oxygen species (ROS), nanotechnology, disease treatment and diagnosis, pyroptosis, aging

In the published article, an author name was incorrectly written as “Wonhaw Lee.” The correct spelling is “Wonhwa Lee.”

The authors apologize for this error and state that this does not change the scientific conclusions of the article in any way. The original article has been updated.

